# Nanozymes towards Personalized Diagnostics: A Recent Progress in Biosensing

**DOI:** 10.3390/bios13040461

**Published:** 2023-04-05

**Authors:** Chitra Padmakumari Kurup, Minhaz Uddin Ahmed

**Affiliations:** Biosensors and Nanobiotechnology Laboratory, Integrated Science Building, Faculty of Science, Universiti Brunei Darussalam, Jalan Tungku Link, Gadong BE1410, Brunei

**Keywords:** nanozyme, point of care, peroxidase, oxidase, SOD, catalase, colorimetric, fluorescence, electrochemical, SERS

## Abstract

This review highlights the recent advancements in the field of nanozymes and their applications in the development of point-of-care biosensors. The use of nanozymes as enzyme-mimicking components in biosensing systems has led to improved performance and miniaturization of these sensors. The unique properties of nanozymes, such as high stability, robustness, and surface tunability, make them an attractive alternative to traditional enzymes in biosensing applications. Researchers have explored a wide range of nanomaterials, including metals, metal oxides, and metal–organic frameworks, for the development of nanozyme-based biosensors. Different sensing strategies, such as colorimetric, fluorescent, electrochemical and SERS, have been implemented using nanozymes as signal-producing components. Despite the numerous advantages, there are also challenges associated with nanozyme-based biosensors, including stability and specificity, which need to be addressed for their wider applications. The future of nanozyme-based biosensors looks promising, with the potential to bring a paradigm shift in biomolecular sensing. The development of highly specific, multi-enzyme mimicking nanozymes could lead to the creation of highly sensitive and low-biofouling biosensors. Integration of nanozymes into point-of-care diagnostics promises to revolutionize healthcare by improving patient outcomes and reducing costs while enhancing the accuracy and sensitivity of diagnostic tools.

## 1. Introduction

Recent years have seen a surge in interest in early disease detection as a new area of medical research due to its apparent ability to reduce mortality rates and raise survival rates. Modern research progress has played a significant role in sustaining high levels of healthcare and general well-being. Although conventional technologies like real-time polymerase chain reaction (RT-PCR) [1,2], enzyme-linked immunosorbent assay (ELISA) [3], high-performance liquid chromatography (HPLC) [4], gas chromatography–mass spectrometry (GCMS) [5], and so on demonstrate high sensitivity and accuracy, the complex and costly equipment required could cause a delay in response time. This highlights the essential need in biosensor and bioassay research for the development of rapid, portable, and user-friendly assays that could accelerate the diagnostic process and alleviate treatment delay [6,7]. Point-of-care (POC) testing is a catch-all word for diagnostic treatments that are conducted directly at the site of a patient. 

Consequently, there has been an effort to develop advanced biosensing technologies that can accurately detect biomarkers, the naturally occurring molecules that indicate the presence of a disease and can be utilized to monitor disease outbreaks and facilitate timely diagnosis [8]. When a patient’s blood, serum, urine, saliva, or tears are placed on a biosensor’s surface, the target biomarker reacts with the bioreceptor (an enzyme, an antibody, a protein receptor, DNA, or whole cells) attached to the sensor, and the presence or absence of disease is determined based on the resulting signal change. Bio-threat agents, chemical contaminants, toxins, bio-molecular targets, and pathogens are all within the detection range of biosensors [9,10,11]. Over the past decade, many biosensors have emerged as viable complementary or alternative detection equipment to traditional methods, allowing for faster and more precise detection in the aforementioned areas of study.

Enzymes play the role of catalysts, speeding up the biochemical processes that take place in the biological molecules [12]. While proteins are typically thought of as enzymes, some RNA molecules have enzymatic activity, and both of them exhibit remarkable efficiency and substrate specificity. By lowering the activation energy needed for a reaction to take place, enzymes speed up chemical processes. Due to their exceptional biocatalytic activity, natural enzymes have been widely used as a key component, the bioreceptor in the development of point-of-care biosensors [13]. The biological component of a biosensor interacts with the target analyte to provide a signal that is proportional to the concentration of the analyte. The transducer turns this signal into a measured output that can be shown on a readout or sent to a computer for analysis [9]. Natural enzymes such as horseradish peroxidase (HRP) [14], glucose oxidase [15], alcohol oxidase [16], lactate oxidase [17], cholesterol oxidase [17], cytochrome c reductase [18], and acetylcholinesterase [19] have been used as the bioreceptor in biosensing applications. Variations in environmental factors, such as temperature, pH, and ionic strength, can diminish the enzyme’s stability and activity over time [20]. Enzymes can also be difficult to store effectively and may require specialized storage conditions to preserve their activity and they may also have a short shelf life, leading to a brief lifespan for biosensors that employ them. Enzymes may also be susceptible to interference from other substances in the sample, resulting in either false-positive or false-negative results [19]. Natural enzymes have limited use in fields such as biomedicine, environmental protection, biosensing, and food processing due to the aforementioned limitations. As a result, researchers have devoted enormous resources to the study of artificial enzyme mimics to circumvent these restrictions. Current developments in nanotechnology have led to the development of functional nanomaterials with natural enzyme-like activity. These nanomaterials are called “nanozymes” because they mimic the catalytic action of enzymes. Nanozymes can perform the same kinetic behaviors as natural enzymes and catalyze the conversion of substrates to oxidized coloring products [21,22]. In 2004, Pasquato and coworkers coined the term “nanozymes” to represent the ribonuclease-mimicking activity of triazacyclononane functionalized gold nanoparticles (NPs) in the transphosphorylation reaction. Numerous nanomaterials have been discovered to have biocatalytic properties since the first nanozyme (Fe_3_O_4_ NPs) was discovered [23]. For the oxidation of chromogenic substrates like o-phenylenediamine dihydrochloride (OPD), 3,3′-diaminobenzidine (DAB), and 2,2′-Azinobis(3-ethylbenzothiazoline-6-sulfonic acid) (ABTS), nanomaterials including metals, metal oxides, carbon nanomaterials (CNMs), and metal–organic frameworks (MOFs) mimic the behavior of natural enzymes [24,25]. Each substrate’s oxidation can produce a different color in aqueous solutions; these solutions can be examined visually, and the absorption spectrum can be identified with a spectrophotometer. Nanozymes are often seen as functionally equivalent replacements for natural enzymes because of their customizable catalytic activity, adaptability, surface area, cost, and manufacturing scale. As a result of their unique features, nanozymes can also serve as recognition receptors [26] or signal tags [27]. Signal amplification by nanozymes has allowed for improvements in the performance and sensitivity of a wide variety of biosensor platforms, including colorimetric, fluorometric, chemiluminescent, surface-enhanced Raman scattering, and electrochemical biosensors [21]. Up until now, there has been a dearth of comprehensive reviews on the use of nanozyme-based biosensors, particularly as they relate to personalized diagnostics. This article aims to address that gap by providing a detailed and comprehensive overview of the use of nanozyme-based biosensors in POC settings. In doing so, we aim to explore the catalytic mechanisms employed by nanozymes for biosensing and provide an overview of the various types of nanozyme-based biosensors currently in use.

## 2. Classification of Nanozymes

**Figure 1:**
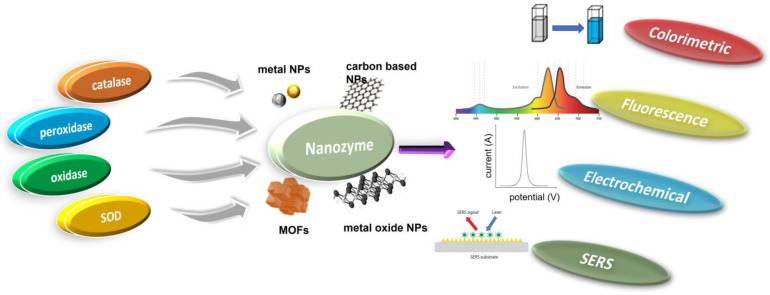
Schematic illustration of various mimicking types of enzymes and the nanozyme base.

Natural enzymes play a crucial role in the biochemical processes that sustain life, but they also have some significant limitations that make it important to investigate potential substitutes [28]. Numerous nanomaterials have been proposed as possible enzyme candidates for practical applications by researchers. Although other characteristics such as size, shape, coating, surface modification, pH, and temperature can have a major influence, the atomic composition of nanozymes is the most relevant since the atoms on the surface and inside the core of the NPs are responsible for the enzymatic activity of the nanozyme [29,30]. Therefore, the incorporation of various NPs may either modify the basic characteristics of nanozymes or provide for their multifunctionality. According to their enzymatic activity, nanozymes fall into two broad categories: oxidoreductase and hydrolase. Family members of the oxidoreductase class perform redox catalysis, much as catalase, superoxide dismutase (SOD), oxidase, peroxidase, and nitrate reductase. Similar to phosphatases, proteases, nucleases, esterases, and silicatein, hydrolases catalyze hydrolysis processes [31]. Nanozymes based on peroxidase, superoxide dismutase (SOD), catalase, and oxidase are commonly used in biosensing applications [32,33,34,35,36]. Recent years have seen the publication of a variety of articles addressing the topic of nanomaterial-based enzyme mimics, with subjects from peroxidase mimics and oxidase mimics to catalase mimics and sulfite oxidase mimics [25,26,29,36,37]. However, the purpose of this review is to provide readers with an idea of the state of the art in the burgeoning field of nanozyme-based biosensors. Since recent developments in chemical synthetic methods have led to the formation of nanomaterials with precise controls of size, shape, and compositions, we hope this review article can highlight the various new nanomaterials used and thereby facilitate the research in enzyme mimics. In this section, we explore the plethora of nanomaterials that exhibit these enzyme-mimicking properties, as well as the method by which they function in biosensing applications (Figure 1).

### 2.1. Peroxidases

**Figure 2:**
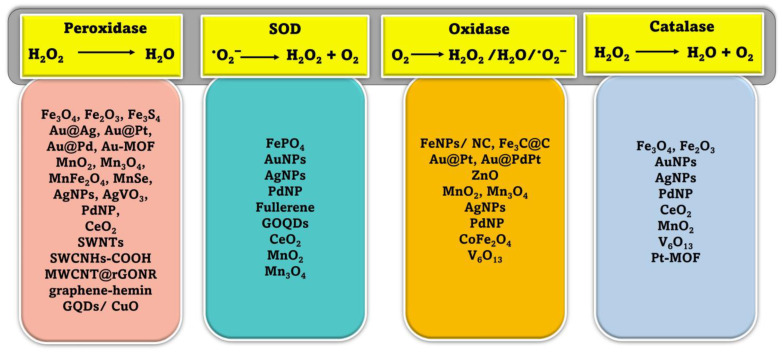
Schematic representation of different enzyme-mimicking actions of nanozymes with examples [24,52,53].

Peroxidases are a broad family of isoenzymes found in various sources, such as plants, animals, and microbes. They generally contain an iron-porphyrin derivative (heme) in their active site, which can accelerate biological oxidation events. In these reactions, the organic hydroperoxides or hydrogen peroxide act as electron acceptors and collaborate with oxidized redox substrates, which serve as electron donors during the reduction process. (Figure 2). The ping-pong mechanism is the recognized mechanism for peroxidase activity (double-displacement reaction). There are two common catalysis pathways for nanozymes in a peroxidase mimic reaction: an electron transfer pathway and a hydroxyl radical production pathway based on the Fenton reaction [38]. The Fenton reaction is mainly responsible for the peroxidase-like activity of nanomaterials [39]. In the Fenton process, the hydrogen peroxide (H_2_O_2_) is catalytically broken down by the ferrous ion (Fe^2+^) in a sequence of reactions in solution (summarized by Equations (1)–(3), leading to the generation of reactive oxygen species [40].

Fe^2+^ + H_2_O_2_ + H^+^ → Fe^3+^ + ^•^OH + H_2_O (1)

Fe^3+^ + H_2_O_2_ → Fe^2+^ + ^•^OOH + H^+^ (2)

Fe^3+^ + OOH → Fe^2+^ + O_2_ + H^+^ (3)

In 2007, it was demonstrated that magnetic nanoparticles made of iron oxide (Fe_3_O_4_) possess the ability to catalyze the oxidation of TMB, o-phenylenediamine (OPD), and diazoaminobenzene (DAB) in the presence of H_2_O_2_ under acidic pH conditions. The resulting reaction produced a range of colored products, such as blue, orange, and brown, resembling the outcomes of the natural enzyme HRP [23]. More recently, an electron transfer pathway has also been identified to increase the peroxidase activity [41]. Their peroxidase-like activity was determined by the number of oxidized products produced and H_2_O_2_ consumed. As possible peroxidase (POD) mimics, transition metal dichalcogenides (TMDs) are a type of 2D materials with considerable potential. Several properties of PODs, such as their active edge locations and surface electron transfer capabilities, contribute to this. TMDs, such as molybdenum disulfide (MoS_2_), tungsten diselenide (WSe_2_), and tungsten ditelluride (WTe_2_), have comparable active sites and electron transfer abilities to PODs [42,43,44]. The maximum turnover number (K_cat_), maximum reaction speed (V_max_), and Michaelis–Menten constant (K_m_) are determined for enzyme kinetics studies using the Michaelis–Menten equation. In addition to the Fenton reaction, H_2_O_2_ can be transformed into reactive hydroxyl radicals (OH) and superoxide anion (O_2_) via “Haber–Weiss reactions” in the presence of strong catalytic metal ions (often iron ions) [45]. Nanozymes made of metallic NPs have numerous applications. The detection of inherent peroxidase activity in Fe_3_O_4_ nanoparticles [23], which closely resembles the peroxidase system found in nature (specifically, the horseradish peroxidase enzyme), has inspired the exploration of peroxidase-mimicking biosensors based on nanomaterials. These biosensors have received significant attention over time [46]. CNMs possess several appealing characteristics as peroxidase mimics, including a high specific surface area, high water solubility, stability, biocompatibility, and non-toxicity [47]. It was shown that single-walled carbon nanotubes (SWCNTs) have peroxidase-like activity, just as natural HRP [48]. Similar to HRP, SWNTs catalyze the peroxidase substrate 3,3,5,5-tetramethylbenzidine (TMB), resulting in a change in color that is highly sensitive to changes in pH, temperature, and H_2_O_2_ concentration. These intriguing results have encouraged the study of different carbon-based nanomaterials as peroxidase mimics in the field of biosensors, including GQDs [49], carbon dots [50], and graphitic carbon nitride [51].

### 2.2. Superoxide Dismutase (SOD)

When it comes to protecting mammalian cells from damage, natural SOD enzymes are indispensable because of their ability to catalyze the dismutation reaction that converts superoxide anion O_2_ (one of the reactive oxygen species, (ROS)) into harmless hydrogen peroxide (H_2_O_2_) and oxygen (O_2_) (Figure 2). Increased oxidative stress and negative health impacts may result from defective deregulation of ROS formation at the cellular level [54]. Some inorganic NMs have been shown to have scavenging ^•^O_2_^−^ activity, similar to that of SOD, and mostly they are more reliable and less expensive than SOD [55,56]. Additionally, unlike SOD, these nanozymes exhibit both electrical and magnetic properties. Therefore, NMs with SOD-like catalytic activity have shown the potential to develop novel biosensors in various areas [53,56]. While these NMs have some catalytic activity toward removing ^•^O_2_^−^, it is significantly less potent than that of SODs, which limits their utility [57]. In recent years, there have been multiple attempts to develop a highly effective nanozyme that can mimic the behavior of superoxide dismutase (SOD). After discovering fullerene’s radical sponge properties, researchers began using it and its derivatives to prevent oxidative damage to neurons [58]. The water-soluble C_60_ fullerene was proposed to catalyze the removal of ^•^O_2_^−^ in two stages. In the presence of protons in the solution, the catalyst is reduced by taking one electron from ^•^O_2_^−^, and then that electron is transferred to another ^•^O_2_^−^, resulting in the production of ^•^O_2_^−^ and H_2_O_2_ [59]. The ceria nanoparticles (CeNPs) are the most researched of the nanozymes that mimic SOD. CeNPs mimicking SOD activity with enhanced catalytic efficiency were originally reported by the Self group [60]. Numerous studies have indicated that the ability of CeNPs to mimic the behavior of superoxide dismutase (SOD) is primarily linked to the presence of an electron shuttle between their mixed oxidation states, which consist of Ce^3+^ and Ce^4+^ [61].

### 2.3. Oxidase Mimics

Enzymes known as natural oxidases can facilitate the oxidation of a substrate, or electron donor, to produce its corresponding oxidized product in the presence of oxygen, typically resulting in the formation of H_2_O, H_2_O_2_, or ^•^O_2_^−^, as shown in Figure 2. Recent research has shown that a variety of NMs are capable of catalyzing the oxidation of single or multiple substrates in oxygen-rich environments, demonstrating properties that are identical to those of natural oxidases [33,62,63].

To evaluate the oxidase-like activity of different nanomaterials, organic substrates containing amino groups, including polyamines and aromatic amines, as well as TMB, OPD, and ABTS, are commonly used because they produce a noticeable change in color and a sensitive signal response when exposed to UV-visible light. A variety of biomedical uses for citrate-capped Au NPs have prompted much research. Extensive research has been conducted on the catalytic properties of “naked” or citrate-capped gold nanoparticles (Au NPs). Rossi and colleagues showed that water-dispersed gold sol may facilitate the oxidation of beta-D-glucose by O_2_ without the need of conventional supports like carbon or protectors such as PVP [64]. Contrary to what could be expected based on control tests, other metal nanoparticles did not exhibit pronounced catalytic ability on the glucose oxidation reaction. It was discovered, in 2009, that nanoceria, which contains a Ce^3+^/Ce^4+^ redox pair, could catalyze the oxidation of organic molecules in ambient conditions [65]. Adsorption of O_2_ is preferentially favored by nanoceria defect sites. This results in the oxidation of TMB and the reduction of Ce^4+^ to Ce^3+^ on the nanoceria surface from the adsorbed O_2_. Afterwards, the newly formed ^•^O_2_^−^ will re-oxidize the Ce^3+^ to Ce^4+^. Nanoceria’s oxidase-mimetic properties can be traced back to the redox switching of Ce^3+^/Ce^4+^ and the production of ^•^O_2_^−^ radicals [66]. Nanomaterials based on manganese (Mn) were also frequently described as acting as oxidase mimics (MnO_2_ and Mn_3_O_4_). An example of a nanomaterial with oxidase-like properties is MnO_2_ nanoparticles, which have been shown to promote the oxidation of substrate molecules such as TMB and OPD using O_2_, resulting in a color change reaction [67].

### 2.4. Catalase Mimics

The ability of natural catalase enzymes to catalyze the cellular degradation of H_2_O_2_ into the water and molecular oxygen is of great importance (Figure 2). An abundance of nanomaterials, including metals and metal oxides, showed catalase-like activity. In most cases, the reported nanomaterials contained catalase-like activities in addition to other enzyme-mimicking activities, and the main enzyme-mimicking activity depended on the pH or temperature [68]. It is vital to remember that if H_2_O_2_ levels are not monitored closely, it can contribute to the spread of several different diseases. Consequently, catalase enzymes are crucial for getting rid of H_2_O_2_ buildup in the cytoplasm by dismutating it into harmless water and oxygen molecules. Researchers have discovered a wide variety of metal-based nanomaterials (Au, Ag, Pd, Pt) [69] and metal oxide-based NPs (cerium oxide, iron oxides, and cobalt oxide nanoparticles) [69,70,71] displaying catalase enzyme-like activity in recent years. Most nanomaterials had catalase-like and other enzyme-mimicking properties. The catalytic reaction’s pH and temperature determine whether these switchable enzyme-mimicking features coexist [69]. Metal nanoparticles can act as mimics of different enzymes depending on the conditions they are in. Under basic pH conditions, they can act as mimics of catalase, which decomposes hydrogen peroxide (H_2_O_2_) into water (H_2_O) and oxygen gas (O_2_). However, in acidic pH conditions, certain metal nanoparticles can exhibit peroxidase-like activity, similar to the natural enzyme horseradish peroxidase, which catalyzes the oxidation of substrates, with hydrogen peroxide as the oxidant [72].

## 3. Nanozyme-Based Biosensors

The use of nanozymes for developing biosensors that can mimic enzyme-like catalytic activity and amplify signals has gained popularity in recent times. They have been found to be an excellent substitute for biological enzymes in the fabrication of innovative biosensors. We discuss the wide range of nanozyme-based biosensors that have been developed and utilized successfully, employing various techniques, such as colorimetry, fluorescence, electrochemistry, surface enhanced Raman, and scattering (Table 1).

### 3.1. Colorimetric Biosensors

As a promising technique for point-of-care detections, colorimetric biosensors allow for the quantitative identification of a specific analyte by color changes using only one’s eyes or a simple portable optical detector. A catalyst’s catalytic activity may also influence the sensing efficiency. Natural enzymes are often used in colorimetric detection methods due to their great sensitivity and specificity [92]. However, their utilization is constrained by inherent restrictions such as high cost, complex treatments, low stability, and challenging storage. Nanozymes, which combine the benefits of natural enzymes with those of nanomaterials, have gained a lot of attention as a viable alternative to natural enzymes because of their low synthesis cost, ease of recycling, and the aforementioned advantages [10]. Enzymes and nanozymes provide colorimetric output signals when they react with chromogenic substrates like 3,3′,5,5′-tetramethylbenzidine (TMB), 2,2′-azino-bis (3-ethylbenzothiazoline-6-sulfonic acid) diammonium salt (ABTS), and o-phenylenediamine (OPD) [21]. We have chosen a few publications to serve as examples of the mechanism of nanozyme-based colorimetric biosensors.

**Figure 3:**
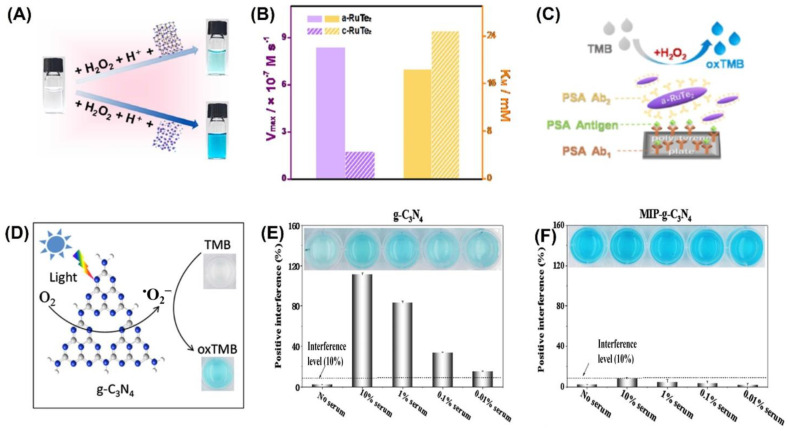
(**A**) RuTe_2_ nanorods demonstrate peroxidase-like activity by catalyzing the oxidation of TMB similar to POD. (**B**) Comparison of the enzymatic parameters V_max_ and K_M_ of amorphous and crystalline RuTe_2_ using H_2_O_2_ as a substrate. (**C**) Representation of the colorimetric a-RuTe_2_-ELISA for detecting PSA. (**D**) Illustration of g-C_3_N_4-_catalyzed oxidation of TMB. Experimental conditions: g-C_3_N_4_ concentration of 20 mg/mL, solution pH of 4.0, TMB concentration of 0.5 mmol/L, and blue LED color. Interfering matrices from serum samples are demonstrated in (**E**) g-C_3_N_4_ and (**F**) MIP-g-C_3_N_4_. Reprinted with permission from refs. [96,101].

Colorimetric sensors were developed using a variety of inorganic nanomaterials with peroxidase-like activity, including transition metal oxides (e.g., ferromagnetic nanoparticles, MOFs), metals (nanohybrids of gold nanoparticles and MoS_2_ nanoribbons), and carbon-based nanomaterials (e.g., graphene dots, graphene, and carbon nanotubes). Liu et al. were able to successfully develop a simple and cost-effective approach for the simultaneous detection of three liver-related biomarkers—aspartate transaminase (AST), alanine transaminase (ALT), and alkaline phosphatase (ALP)—from human plasma employing Au-decorated CoAl-layered double oxide (Au/LDO) nanozymes [93]. Two-dimensional (2D) nanomaterial layered double hydroxides (LDHs) are also known as anionic clays or hydrotalcite-like compounds. One of the most researched LDHs, CoAl-LDHs convert between divalent Co^2+^ and trivalent Al^3+^ ions, which could aid in the faradaic redox process [94]. The nanozyme Au/LDO, combined with the agarose hydrogel, acted as a peroxidase mimic, expediting the transformation of colorless 3,3′,5,5′-tetramethylbenzidine (TMB) to blue oxTMB in the presence of hydrogen peroxide (H_2_O_2_). Significant interest has been drawn to the specific advantages of the amorphous structure over the crystalline structure in the realm of catalysis. Improved activity and enhanced catalytic selectivity are two of the benefits [95]. Peroxidase mimetic amorphous ruthenium hexamine/tellurium nanorod (a-RuTe_2_) has a catalytic constant (K_cat_) 3.77 times higher than that of crystalline RuTe (c-RuTe_2_), as indicated by the work of Yan et al. (Figure 3A,B) [96]. Based on this, an enzyme-linked immunosorbent assay (ELISA) was developed for the detection of prostate-specific antigen (PSA) using a-RuTe_2_ nanorods as labels (Figure 3C). The proposed ELISA based on a-RuTe_2_ nanorods has a very high sensitivity, at approximately an order of magnitude lower than that of a standard ELISA based on natural horseradish peroxidase.

Similarly, an ultrasensitive immunosensor was constructed for ApoA1 detection using POD-mimicking nanozymes synthesized from Prussian Blue (PB) and magnetic graphene oxide (MGO, PMGO). By catalyzing the oxidation of a colorimetric substrate, TMB, the produced nanozyme could be used as a signal-generating material [97]. Increasing interest in metal–organic frameworks (MOF) can be attributed to the materials’ many desirable qualities, such as their high specific surface area and pore volume, their malleable composition, and their remarkable thermal stability [98]. Recently, MOF-818, a nanozyme-mimicking catechol oxidase, was homogeneously prepared and characterized on the surface of carbon cloth (CC) fibers using a hydrothermal method. The nanozyme displayed high catalytic activity toward the oxidation of pale yellow 3,5-di-tert-butylcatechol (3,5-DTBC) to bright yellow 3,5-di-tert-butyl-(3,5-DTBQ) [73]. Following aptamer modification on the MOF-818/CC surface, thrombin was detected, which hindered the catalytic activity of the nanozyme composite. The aptamer-MOF-818/CC selectively and sensitively measured thrombin with an LOD of 6.4 pM.

The active surface of nanozymes allows for rapid interaction with a range of biomolecules, from low-molecular-weight compounds to macromolecules. However, interference from the biological sample matrix is a persistent issue. To address this concern, molecularly imprinted polymers (MIPs) have been used as they can create large-scale target molecule-binding sites, reducing interference from other molecules [99,100]. In their research, Wu et al. [101] discovered that the MIP graphitic carbon nitride (MIP-g-C_3_N_4_) nanozyme possessed intrinsic photooxidase activity (Figure 3D), which led to an increase in the enzyme’s bioactivity and target specificity. They also found that this nanozyme reduced matrix interference from serum samples by a factor of 1000 (Figure 3E,F). During colorimetric sensing, MIP-g-C_3_N_4_ displayed enzyme activity that was four times higher than that of bare g-C_3_N_4_. Using the novel nanozyme, the group was able to detect L-cysteine, a biomarker associated with a range of diseases like cancer and Alzheimer’s. Nanozyme-based colorimetric biosensors have proven to be highly useful owing to their ease of use, quick response time, portability, and adaptability. However, their detection accuracy and sensitivity can be compromised by the background color of the sample, which can cause interference [21,22].

### 3.2. Fluorescence Biosensors

Fluorescent biosensors, which consist of small scaffolds, can be attached to molecules using one or more fluorescent probes through enzymatic, chemical, or genetic methods. This technology has emerged as a sensitive and effective approach for biosensing due to its ability to improve sensitivity and reduce matrix effects. The biosensor’s detection function relies on the activation or deactivation of its fluorescence by the target analytes, which can either turn it on or turn it off [102]. Fluorescence is a form of luminescence that occurs when a substance absorbs light with high energy (shorter wavelength) and subsequently emits light with lower energy (longer wavelength). This process occurs rapidly, typically taking place within 10^−8^ to 10^−9^ s [103]. Nanozyme-based fluorescence approaches have been developed for a number of diagnostic applications and have recently attracted a lot of attention in biosensing research. One type of these sensors is fluorescence resonance energy transfer (FRET) biosensors, which rely on the direct excitation of a fluorophore donor by electromagnetic emission at the appropriate wavelength [104]. The substances that prevent excited fluorophores from emitting further light and instead turn that energy into heat are called quenchers. Quenchers are effective energy acceptors in FRET pairs because they maintain their darkness by releasing the absorbed energy as heat.

**Figure 4:**
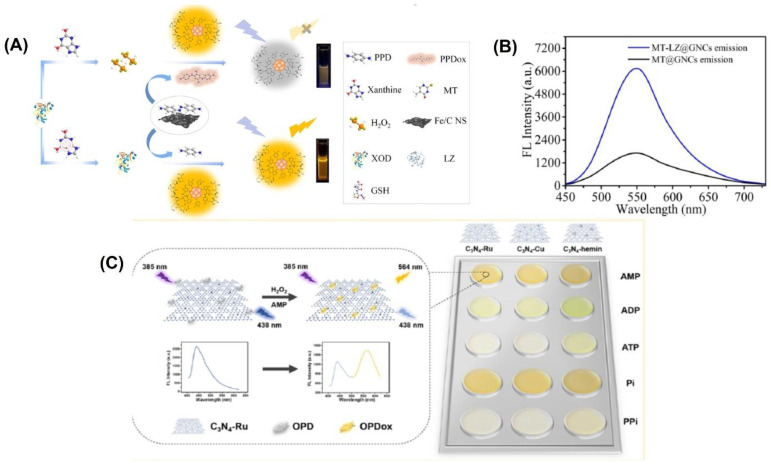
(**A**) Schematic representation of the fabrication of MT-LZ@GNCs. (**B**) Fluorescence emission spectra of MT@GNCs and MT-LZ@GNCs. (**C**) Schematic representation of the principle behind the assay and the color change obtained with the three different materials. Reprinted with permission from refs. [51,105].

For example, gold nanoclusters were modified by Wang et al. with lysozyme-functionalized 5-methyl-2-thiouracil gold nanoclusters (MT-LZ@GNCs) (Figure 4A) to enhance the fluorescence activity. [105]. The MT-LZ@GNCs exhibited a yellow fluorescence when measured at a wavelength of 550 nm (Figure 4B). To create a fluorescent nanoprobe for detecting xanthine, MT-LZ@GNCs was combined with an iron-doped carbon nanosheet (Fe/CNS), which has similar activity to the enzyme peroxidase. The resulting MT-LZ@GNCs/Fe/CNS fluorescent nanoprobe was able to detect xanthine, and it showed a 3.67-fold increase in fluorescence intensity compared to MT@GNCs. The nanoprobe was able to detect xanthine in human serum samples with a low detection limit of 0.23 µmol L^−1^ and a recovery rate between 98.72% and 109.27%.

In another study, a fluorescence biosensor was fabricated based on the dual functions of MIL-101(Fe) particles, peroxidase-mimicking activity and fluorescent emission, for the simultaneous detection of choline and acetylcholine (ACh) [106]. This method of fluorescence sensing uses acetylcholinesterase (AChE) to break down ACh into choline, which is then oxidized by choline oxidase (ChOx) to create H_2_O_2_. The H_2_O_2_ is then broken down into hydroxyl radicals using MIL-101(Fe) nanozymes, resulting in the oxidation of the non-fluorescent terephthalic acid of MIL-101(Fe) to form a highly fluorescent 2-hydroxy terephthalic acid. The MIL-101(Fe) nanozyme has a much greater affinity for H_2_O_2_ than the enzyme HRP, as shown by its Km value being around 67 times lower. This indicates that the MIL-101(Fe) nanozyme is more effective at detecting H_2_O_2_ than HRP. Using this biosensor, choline in milk and ACh in human plasma were detected, with recoveries ranging from 99.63% to 102.00% and 97.20% to 102.91%, respectively.

A ratiometric fluorescence sensing system requires the integration of two emitting components, which can be accomplished through the production of nanoparticles or various organic dye composites and the manufacture of intrinsic dual-emission fluorophores [13,14]. Unfortunately, the general application of such approaches is hampered by the fact that they typically necessitate elaborate and time-consuming pretreatment steps [15]. Graphitic carbon nitrides (C_3_N_4_) have been utilized to create highly active nanozymes for biosensing applications. These nanozymes have abundant pyridinic nitrogen moieties and a π-conjugated framework that provides potential binding sites for further modifications to enhance their catalytic activity. Wang et al. [51] developed three fluorescent C_3_N_4_-based nanozymes, namely C_3_N_4_-Ru, C_3_N_4_-Cu, and C_3_N_4_-hemin, with excellent peroxidase-like activities. They combined ruthenium and copper ions into the nanosheets through coordination with pyridinic nitrogen moieties, while hemin was linked to C_3_N_4_ through π-π interaction. These fluorescent nanozymes emitted a fluorescence at 438 nm when excited at 385 nm. An intriguing observation was made when the nanozymes were present during the catalytic oxidation of o-phenylenediamine (OPD) to oxidized OPD (OPDox) in the presence of H_2_O_2_. In addition to the emission of a new fluorescence at 564 nm by the OPDox, the fluorescence at 438 nm of the nanozymes was also quenched. As a result, the researchers used the ratio between the fluorescent intensity at 564 and 438 nm (F564/F438) as the signal output to create a ratiometric biosensing system. To create a ratiometric H_2_O_2_ sensing system, the C_3_N_4_-Ru nanozyme was used (Figure 4C). In order to detect and differentiate between five phosphates, a ratiometric sensor array was built using three distinct C_3_N_4_-based nanozymes.

Li and colleagues developed a dual-emission ratiometric fluorescence sensing system using MnO_2_ nanosheets (MnO_2_ NSs) as quenchers for blue fluorescent carbon dots (BCDs) to determine multiple H_2_O_2_-related biomarkers with high accuracy and reliability [107]. The system avoids the need for synthesizing dual-emission fluorophores, making it simple, sensitive, and versatile. MnO_2_ NSs possess oxidase-like activity that can convert non-fluorescent o-phenylenediamine (OPD) to 2,3-diaminophenazine (DAP), producing a fluorescence signal at 562 nm. As mediators, MnO_2_ NSs decompose in the presence of H_2_O_2_, resulting in the fluorescence recovery of BCDs and a decrease in DAP. Sarcosine, a prostate cancer biomarker, generates H_2_O_2_ when catalyzed by sarcosine oxidase. Under optimal conditions, sarcosine can be detected as low as 0.36 μM. The system’s practicability was demonstrated by successfully detecting sarcosine in human urine samples with satisfactory recoveries of 94.9–98.6%.

### 3.3. Electrochemical Biosensors

Incorporating the natural bioselectivity of the biological component, electrochemical biosensors combine the sensitivity of electroanalytical methods with the analytical precision of the chemical component. Once the analyte has been recognized by the biological component of the sensor, a catalytic or binding event will follow, resulting in an electrical signal that is measured by a transducer and will be proportionate to the analyte concentration [108,109,110,111,112,113]. Electrochemical biosensors have been widely used in numerous industries, including clinical diagnostics, environmental monitoring, food safety analysis, etc., because of their ease of use, low cost, exceptional stability, and sensitive response [6,114]. Since the nanozymes have a large surface area and a high density of capture sites, they may be able to increase the loading of the electroactive species at their surfaces, leading to better electrochemical reactions [21,29,115]. Thus, nanozymes can serve either as an electrode material [116] or as a tracer tag [117] for signal amplification in electrochemical biosensors. Graphene oxides, fullerenes, carbon nanotubes (CNTs), and AuNPs Zr and Cu-based MOFs are just some of the nanozymes employed in EC biosensors due to their exceptional catalytic activity [10,118].

It was worth noting that Gugoasa’s team developed the first in situ synthesis method for a hybrid material made of gold nanoparticles and reduced graphene oxide (Au-rGO), which combines the unique features of each nanomaterial [119]. Laccase-like catalytic activity was demonstrated by the Au-rGO on a phenolic substrate (catechol). Compared to unmodified SPE 0.073 cm^2^, the electroactive surface area of Au-rGO/SPE was increased to 0.215 cm^2^. Au-rGO’s surface area, efficient electro-catalysis, and high conductivity aided in electron transfer between the analyte and electrode, boosting the response signal of the SPE-modified electrode.

**Figure 5:**
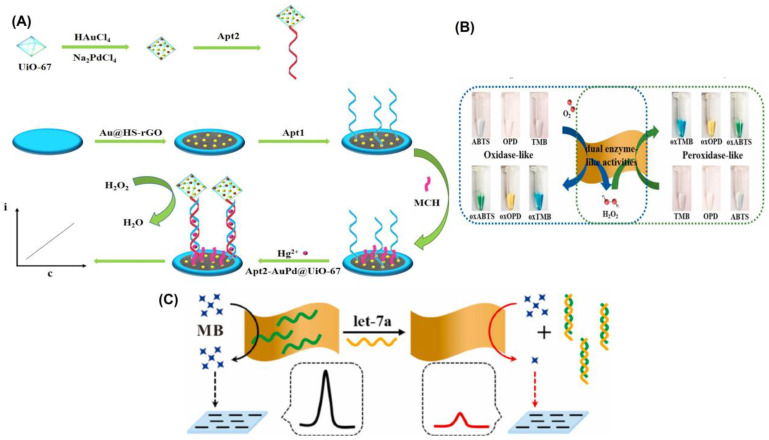
(**A**) Schematic diagram of the synthesis of Apt2-AuPd@UiO-67 and illustration of the construction of the electrochemical aptasensor. (**B**) Graphical representation of 2D MnO_2_ nanoflake interaction with enzymatic substrates through oxidase mimetic and peroxidase mimetic activities. The substrate concentrations of TMB, OPD, and ABTS are all 0.5 mM, and the 2D MnO_2_ nanoflake concentration is 2.6 μg/mL. (**C**) Illustration of the detection concept of a homogeneous electrochemical biosensor for miRNA assay. Reprinted with permission from refs. [32,125].

Recent years have seen a surge in interest in metal–organic frameworks (MOFs) as promising new materials due to their tailorable pore size, functional groups, and biocompatibility. In comparison to previous porous solid supports (such as zeolites, mesoporous silica, sol-gel hydrogels, and porous polymers), MOFs have greatly broadened the possibilities for immobilizing enzymes, and are seen as a highly promising platform for researching enzyme–host material interactions [120,121]. Therefore, for the immobilizing of nanoparticles, they are also widely utilized as a matrix [122]. For the sensitive detection of Hg^2+^, Wang’s team decorated zirconium MOFs with a complex of gold and palladium (AuPd@UiO-67), which served as a nanozyme to amplify the signal [32]. Electrode modification was accomplished by the use of gold-modified thiol graphene (Au@HS-rGO). As part of the platform construction, an Au-S bond was made to the substrate strand (Apt1). Nanozyme AuPd@UiO-67 was used to label Apt2, and it exhibited catalase-like characteristics (Figure 5A). AuPd@UiO-67 nanozyme’s catalytic action toward H_2_O_2_ allowed for the recording of the current signal. It was reported that the designed electrochemical aptasensor for Hg^2+^ has a low detection limit of 0.16 nmol/L and a wide linear range of 1.0 nmol/L to 1.0 mmol/L.

DNA-based homogeneous electrochemical sensing is a novel approach among electrochemical sensors since it enables target identification in a single, diluted solution [123]. Unlike traditional heterogeneous sensors, homogeneous sensors do not require the immobilization of DNA recognition elements, the laborious functionalization of electrodes, or the washing stages [124]. In a dynamic system, where target identification, real-time assay, and regeneration will be conducted in succession, homogeneous electrochemistry provides additional benefits. Nanomaterials with a uniform size and shape can be produced through cost-effective and sustainable wet chemical synthesis, which is guided by a soft template [85]. 2D MnO_2_ nanoflakes were synthesized by this method by Wu and colleagues [125], and their functionality resembled that of oxidase and peroxidase enzymes (Figure 5B). As a result of their enzyme-like properties, 2D MnO_2_ nanoflakes have been shown to be highly active in catalyzing the oxidation of O_2_ into ROS and greatly lowering the differential pulse voltammetry (DPV) peak current by removing methylene blue (MB). More so, 2D MnO_2_ nanoflakes showed off a peculiar reaction to ssDNA. A homogeneous electrochemical 2D MnO_2_ nanoflake-based biosensor for miRNA, let-7a, was created (Figure 5C), with a linear range of 0.4–100 nM and a LOD of 0.25 nM, due to its sensitivity to ssDNA and dual enzyme-like activities. Similarly, Wang et al. [110] proposed a flow homogenous electrochemical microRNA detection devoid of immobilized DNA recognition components and time-consuming electrode functionalization. This article describes the ultrasonic production of a 2D MOF nanozyme with a thickness of roughly 1 nm with peroxidase-like activity. As part of the DNA-based homogeneous electrochemical sensing system, Co-MOF nanozymes were used as ssDNA collectors and signal amplifiers. Even after being subjected to six cycles of regeneration, the system maintained its peak performance. A 0.12 pM LOD and successful detection of the target microRNA in biological samples demonstrated the system’s exceptional long-term stability. As their sensitivity extends over large dynamic ranges, electrochemical biosensors find widespread use in initial, semi-quantitative, and qualitative screening. In spite of their excellent accuracy and practicality, these biosensors still have some ways to go before they are entirely problem-free because of their poor replication and low stability.

### 3.4. Surface-Enhanced Raman Spectroscopy (SERS) Biosensors

**Figure 6:**
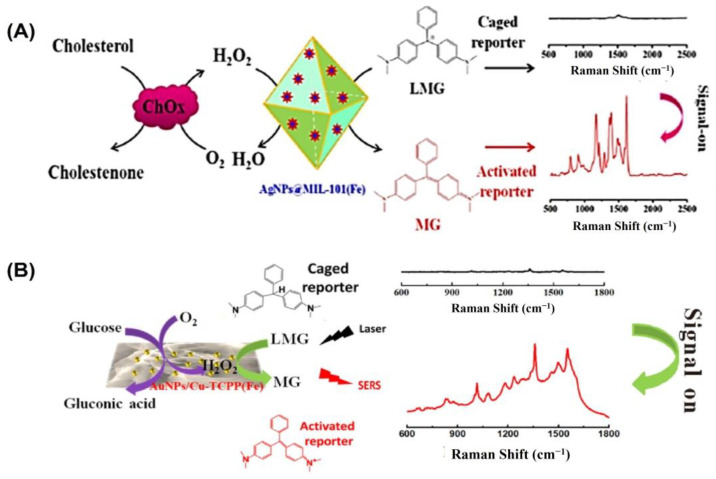
(**A**) Illustration of the cholesterol-detecting SERS biosensor based on AgNPs@MIL-101(Fe). (**B**) Schematic representation of the enzyme-free tandem reaction method for SERS glucose measurement. Reprinted with permission from refs. [87,88].

In the field of bioanalysis and detection, surface-enhanced Raman spectroscopy (SERS)-based biosensors have recently attracted a lot of interest due to their high sensitivity, efficiency, accuracy, low sample demand, and non-destructive nature [126,127]. Raman spectroscopy receives its signal from Raman scattering produced by a Raman reporter on the surface of plasmonic nanomaterials like gold, silver, silicon, porous alumina, or their combinations [128]. The SERS signal can be modified through the reaction of reporter molecules by incorporating peroxidase nanozymes with the plasmonic nanostructures [129]. For example, Wu and colleagues grew silver nanoparticles (AgNPs) on the surface of a metal–organic framework (MOF) named MIL-101 (Fe) to develop a peroxidase-mimicking nanozyme AgNPs@MOF [88] (Figure 6A). The high adsorption capacity of MOFs makes them suitable for usage as SERS substrates. The AgNPs@MOF were used as a substrate for surface-enhanced Raman spectroscopy (SERS) and as peroxidase mimics to convert inert leucomalachite green to Raman-active malachite green. A sensitive and precise SERS sensing platform was proposed for cholesterol monitoring based on the strong peroxidase-mimicking activity and high SERS enhancement of AgNPs@MOF. The constructed biosensor was able to detect concentrations as low as 0.36 µM under the best possible conditions, with a dynamic detection range of 1.0–100 µM.

Likewise, Hu and colleagues developed a non-invasive method of measuring blood glucose levels in saliva [87]. Metalloporphyrin-based metal–organic frameworks are a recently discovered class of peroxidase mimic enzymes that can catalyze the interaction between hydrogen peroxide and 3,3′,5,5′-tetramethylbenzidine (TMB) to generate oxidized TMB (oxTMB). Incorporating AuNPs into MOF-based hybrid nanomaterials has been shown to improve their performance in SERS measurement (Figure 6B). Susceptible to water but not sinking, after synthesizing water-stable 2D metalloporphyrin Cu-TCPP(Fe) nanosheets, hybrid nanosheets of AuNPs/Cu-TCPP(Fe) were formed through in situ growth. It was found that in the presence of oxygen, glucose may be oxidized catalytically by AuNPs, yielding gluconic acid and H_2_O_2_. On the other hand, the Cu-TCPP(Fe) nanosheets can act as a catalyst for transforming oxide-caged leucomalachite green (LMG), which lacks Raman activity, into MG by utilizing H_2_O_2_ that is generated on-site, resulting in a Raman-active signal. GOx-like activities were observed in “naked” gold nanoparticles (Au NPs) that were prepared without stabilizers or protectors [64]. However, it has been observed that the usage of protectors may prevent GOx-like activity. Peroxidase-like activities and SERS activities were both found to be high in Ag nanoparticles [130]. To catalyze the sequential oxidation of glucose, Xia’s group has created synthetic tandem nanozyme Au@Ag NPs that mimic the actions of both GOx and peroxidase [131]. In the realm of surface-enhanced Raman scattering (SERS), core–shell plasmonic nanostructures have recently emerged as an exciting new area of study [132,133]. Shell-isolated nanoparticle-enhanced Raman spectroscopy (SHINERS), which uses plasmonic nanoparticles (NPs) as the core and inert or semiconductor materials as the shell, has emerged as a promising and potent technique in a wide range of chemical and biological investigations. Some of the most promising SHINERS built for biological research, including biosensing, imaging, and treatment, are graphene-isolated metal nanoparticles (GIMNs). Jin’s group designed a monodispersed Ag/oxidized GQDs (o-GQDs) nanohybrid with a small core–shell configuration (ca. 10 nm). Small Ag/o-GQDs have improved biocompatibility and good nucleus-mitochondria dual-targeting capacity without change to the targeting ligand, presenting unparalleled potential for intracellular applications. In addition, the SERS-active Ag/o-GQDs demonstrate a peroxidase (POD)-like response, allowing accurate subcellular detection of intracellular H_2_O_2_ [134].

## 4. Market Opportunities and Commercialization

Bringing nanozyme-based biosensors to the market is a challenging process that involves considerable industry input and market analysis. Despite the clear potential benefits of these biosensors, such as personalized medicine and cost savings, it may take up to a decade to commercialize a biosensor and another decade or more before it becomes profitable. Biosensors have to adhere to a number of different regulations and requirements before they can be commercially supplied, and this is a costly and time-consuming process. It may be difficult to reliably and consistently scale up the manufacturing of biosensors built in the lab to industrial levels. Furthermore, researchers may have limited access to real samples and limited expertise in commercialization, both of which might make validating novel biosensor concepts developed in the lab difficult in the real world. Competing technologies and conventional platforms have lower development costs and a guaranteed market share, due to their longer experience in product formulation. However, advances in micro- and nano-systems, such as polymers and hydrogels, and 3D printing manufacturing, have led to a surge in interest in these technologies despite their higher development costs. Investors have shown a willingness to invest in the healthcare industry when the benefits of new technologies, such as shortened assay times, improved specificity, and point-of-care testing, offset the high research costs. The profitability of nanozyme-based biosensor R&D is also dependent on the supporting technologies and infrastructure. To conclude, successfully commercializing nanozyme-based biosensors requires a comprehensive understanding of market trends, development of enabling technologies, and significant industry involvement. Although there are challenges, the potential benefits of these biosensors make them a promising area for research and development in the healthcare industry.

## 5. Challenges in the Development of Personalized Biosensors

Personalized biosensors provide comprehensive data on an individual’s health and physical activities. This promising development is made possible by the superior precision and robustness of detecting biomarkers in the body [135]. To better translate and use personalized biosensors, there has been an increase in the usage of collaborative system design, which brings together user-defined needs with technological innovation. Moreover, the utilization of personalized biosensors is expanding as sensor technologies progress beyond the conventional biomarker classes of nucleic acids and proteins to encompass metabolites and direct detection of infections. Nanozymes have several properties that make them attractive for personalized diagnostics. Firstly, (a) nanozymes can mimic the catalytic activities of enzymes and can be designed to have high selectivity and sensitivity towards specific biomolecules. This allows for the detection and quantification of biomarkers in biological samples, which can provide valuable information about an individual’s health status. Additionally, (b) nanozymes are highly stable and robust, making them ideal for use in point-of-care diagnostic devices. This could enable real-time monitoring of biomarkers, allowing for early detection and intervention in diseases. Furthermore, (c) nanozymes can be engineered to have unique surface properties, which can enable their integration into personalized diagnostic platforms. For example, nanozymes can be functionalized with targeting moieties such as antibodies or aptamers, allowing for the selective detection of specific biomolecules in complex biological matrices.

Despite significant progress, designing personalized biosensors still faces critical challenges and technological gaps that need to be addressed. One of the biggest challenges is the extremely complex nature of biological samples. Sample matrices can contain a diverse range of non-target compounds at varying concentrations, which can lead to inaccurate detection of the target analyte, either overestimating or underestimating its concentration. This can be further complicated by sensor fouling, especially when the sample matrix includes proteins. Detecting both low and high concentrations with a single sensor presents a significant challenge. As a result, it may be necessary to design multiple sensors to cover the full concentration range. Another option is to create a sensor that functions optimally in the medium-to-low concentration range, with the caveat that samples with higher analyte levels will need to be diluted. As the sample gets more diluted, it becomes increasingly important to consider the sensing threshold.

## 6. Conclusions and Outlook

Clinical diagnosis specialists and medical offices are not the only ones who need these simple and cutting-edge tools; regular people using them in their homes or the field with limited resources also need them. When compared to using natural enzymes as key functional components for analyte detection, the application of nanomaterials exhibiting enzyme-mimicking activity (nanozymes) incorporated in POC-based biosensor systems shows various advantages. This review has covered a wide range of topics pertaining to the use of nanozymes for biosensor creation. The most up-to-date examples of each kind of nanozymes used in POC biosensor development are summarized in this article. Recent advances in these instances show that the study of nanozymes and their biosensing applications has increased steadily. The huge potential of nanozymes in POC-based biosensor development, however, has yet to be realized because of various research gaps and hurdles that have been recognized and need to be handled at this frontier. To produce high-performance nanozymes, it is essential to comprehend their catalytic mechanism and the structure–activity relationship. This understanding can be achieved by combining experimental and computational research. Despite testing several nanomaterials for their ability to imitate natural enzymes, peroxidase mimics, primarily redox enzyme mimics, are still the most popular choice for biosensing. Furthermore, understanding the catalytic mechanism of nanozymes is crucial in determining their efficacy. Given the diversity of natural enzymes, more work needs to go into engineering nanozymes with new catalytic capabilities like synthetase and hydrolase. Similarly, enzyme-active sites are the primary focus of current research. The protein scaffold of an enzyme is crucial to the selectivity and effectiveness of an enzymatic reaction, yet its analogues have not been well investigated. This might be accomplished by taking a page out of the book of building new functional proteins and combining experimental methods with computational and/or theoretical strategies for designing nanozymes. If this were to happen, not only would the versatility of nanozyme applications in biosensor development be greatly increased, but the cost of detecting a wider variety of analytes would also be reduced. Substrate specificity is a vital characteristic of natural enzymes, and the reduced specificity of nanozymes poses a significant challenge. Combining natural enzymes with nanozymes may help mitigate this issue, but it could compromise the stability and cost of the entire catalyst system. While nanozymes show promise as an enzyme replacement, further study is needed before they can compete with normal enzymes. Since chemical processes are typically employed to generate nanozymes, it is essential to increase batch-to-batch reproducibility if these products are to be used in industrial or clinical contexts. Nanozyme-based point-of-care (POC) biosensors will only be widely used if scientists can design them to be easy to use, highly automated, and require minimal user input. Scalability, mobility, and ease of application are all crucial early design considerations that must be made before mass manufacturing can begin.

## Figures and Tables

**Table 1:** Different biosensors based on nanozymes used in biosensing applications.

Nanozyme	Enzyme Mimetic Activity	Transduction System	Nanozyme Activity	Bioreceptor	Application	Limit of Detection	Range	Ref.
MOF-818 on carbon cloth (CC) fibers	catechol oxidase	colorimetric	MOF-818/CC effectively mimicked catechol oxidase by catalyzing 3,5-Ditert-butylcatechol (3,5-DTBC) colorimetric substrates in the presence of oxygen.	aptamer	thrombin	6.4 pM	1.4 × 10^−10^–1.4 × 10^−5^ M	[73]
Bimetallic ZnO-Co_3_O_4_ (zinc oxide/cobalt oxide) nanocages	peroxidase	colorimetric	Due to the high binding capacity of Aβ monomer to ZnO-Co_3_O_4_ NCs, the peroxidase activity of ZnO-Co_3_O_4_ NCs was reduced, and as a result, the TMB color change was lowered as well.		amyloid-β peptide (Aβ)	3.5 nM	5 to 150 nM	[74]
COS (chitosan oligosaccharide)-AuNPs@Fe_2+_	peroxidase	colorimetric	In the presence of PS, COS bind to AuNPs@Fe^2+^ to generate a nanozyme with improved peroxidase-mimicking activity towards TMB.	aptamer	phosphatidylserine (PS)	5 × 10^−10^ mol L^−1^	5 × 10^−7^ mol L^−1^ to 5 × 10^−3^ mol L^−1^	[75]
Ferrite nanozyme	peroxidase	colorimetric	Ferrite nanozyme with improved peroxidase-mimicking activity oxidized TMB to create a color change.		l-cysteine	0.119 μM	0.2–20 μM	[76]
Co–N-C (Co, N co-doped porous carbon) nanozyme	oxidase	fluorescence	The chromogenic substrate TMB was catalyzed and oxidized by Co–N-C nanozyme		butyrylcholinesterase (BChE)	0.16 U L^−1^	0.5 to 40 U L^−1^	[77]
Cu-MOF	peroxidase	fluorescence	TMB was oxidized by Co–N-C nanozyme, and oxTMB caused the quenching of carbon quantum dots fluorescence.	aptamer	C-reactive protein	40 pg mL^−1^	0.1 to 50 ng mL ^−1^	[78]
Cu-MOF	peroxidase	fluorescence	CRP-specific RNA adsorbed on Cu-MOFs inhibited the enzymatic activity and fluorescence of the MOF.	aptamer	thrombin	110 fM		[79]
CuAA nanozyme (copper-doped carbon-based)	peroxidase	fluorescence	CuAA oxidised the OPD into 2,3-diaminophenazine (DAP). DAP further quenched Mg–N-CQDs’ (Mg/N doped CQDs) fluorescence.		glucose	1.56 μM	2–400 μΜ	[80]
Cu (copper)-MOF	peroxidase	fluorescence	The immobilized thrombin-based aptamer on the Cu-MOF surface forms a functionalized composite, ssDNA/MOF, which inhibited the stimulated fluorescence emission and enzymatic activity of Cu-MOF.		pyrophosphatase pyrophosphate ion	0.30 mU mL^−1^ 0.53 μmol L^−1^	2–40 mU mL^−1^ 1 to 450 μmol L^−1^	[81]
Fe_3_O_4_ nanozyme with copper (II) complex	peroxidase	electrochemical	The Fe_3_O_4_ nanozyme functioned as a carrier for hairpin capture probes (HCP) and also amplified the signal amplification through the catalytic reaction.		microRNA	33 aM	100 aM to 100 nM	[82]
Hollow Pt–Fe_3_O_4_@C nanospheres	oxidase	electrochemical	Fe_3_O_4_ hollow nanospheres were used as carriers for Pt, and the combination with carbon (Pt–Fe_3_O_4_@C) achieved high conductivity.		sarcosine	0.43 μM	0.5–60 μM	[83]
CuO	peroxidase	electrochemical	CuO nanozyme functioned as signal amplifying nanoprobes.	aptamer	MCF-7 circulating tumor cell (MUC-1)	27 cells mL^− 1^	50 to 7 × 103 cells mL^−1^	[84]
2D MnO_2_ nanoflakes	oxidase/ peroxidase	electrochemical	MnO_2_ nanoflakes exhibit superior response to ssDNA binding and showed high catalytic activity.		microRNA (let-7a)	0.25 nM	0.4 to 100 nM	[85]
COF@Pt (covalent organic framework-based nanozymes)	peroxidase	electrochemical	COF@Pt functions as peroxidase mimic to amplify the electrochemical response from H_2_O_2_ reduction.		circulating tumor cells	1 cell mL^−1^	2 to 105 cells mL^−1^	[86]
AuNPs/Cu-TCPP(Fe) (Cu-tetra(4-carboxyphenyl)porphyrin)chloride(Fe(III)))	peroxidase	SERS	Cu-TCPP(Fe) nanosheets catalyzed H_2_O_2_ and they further oxidized the non-Raman-active leucomalachite green (LMG) into the Raman-active malachite green (MG)		glucose	0.16 mmol/L		[87]
AgNPs@MOF	peroxidase	SERS	The AgNPs@MOF served as the SERS substrate and as peroxidase mimic to convert the non-Raman-active LMG into Raman-active MG.		cholesterol	0.36 μM	1.0 to100 μM	[88]
Magnetic ring-like -Fe_3_O_4_/Au	peroxidase	SERS	Fe_3_O_4_/Au performed as SERS substrate for detecting the Raman signals of oxidized products.		glutathione cholesterol	0.10 μM 0.08 μM	1 to 150 μM 1 to 100 μM	[89]
PANI@MoS_2_@Fe_3_O_4_@Au (polyaniline@MoS_2_@Fe_3_O_4_@Au)	peroxidase	SERS	PANI@MoS_2_@Fe_3_O_4_@Au nanozymes greatly increased the peroxidase-like activity and the SERS performance.		glucose	10^−12^ M	10^−11^–10^−3^ M	[90]
Au/CoFe_2_ MOF (gold nanostars and metal–organic frameworks)	peroxidase	SERS	Peroxidase-like activity of Au/CoFe_2_ MOFs promotes the formation of OH radicals, resulting in the catalytic oxidation of TMB and the enhancement of the SERS signal.		nicotinamide adenine dinucleotide	28 pM		[91]

## Data Availability

No data were used for the research described in the article.
